# Effects of Acupoint Application on Lower Urinary Tract Symptoms in Patients Undergoing Intravesical Instillation After Bladder Cancer Surgery: A Randomized Crossover Exploratory Study

**DOI:** 10.1155/prm/5079749

**Published:** 2026-10-09

**Authors:** Lei Shi, Cong Chen, Lian Li, Junxiao Chen, Yongtai Zhang, Jianhong Li, Zhijuan Xu, Di Lu, Yingxue Ma, Yuechen Feng, Chenggen Jin, Yaxuan Wang

**Affiliations:** ^1^ Department of Urology The Second Hospital of Hebei Medical University Shijiazhuang China hebmu.edu.cn; ^2^ Nursing Department The First Hospital of Hebei Medical University Shijiazhuang China hebmu.edu.cn; ^3^ Department of Traditional Chinese Medicine The Second Hospital of Hebei Medical University Shijiazhuang China hebmu.edu.cn; ^4^ Department of Hepatobiliary Surgery The Second Hospital of Hebei Medical University Shijiazhuang China hebmu.edu.cn

**Keywords:** acupoint application, intravesical instillation, lower urinary tract symptoms, non–muscle-invasive bladder cancer, quality of life

## Abstract

**Objective:**

Patients with non–muscle‐invasive bladder cancer (NMIBC) often experience lower urinary tract symptoms (LUTS) after postoperative intravesical instillation, impairing quality of life and treatment adherence. This study explored the effects of traditional Chinese medicine (TCM) acupoint application on postinstillation LUTS and quality of life.

**Methods:**

In this randomized, single‐blind, crossover exploratory study, patients receiving postoperative intravesical instillation alternately underwent conventional instillation or instillation following TCM acupoint application, separated by a 1‐week washout period. Primary outcomes included visual analog scale (VAS) scores and 24‐h voiding diary parameters. Secondary outcomes included EORTC QLQ‐C30 and FACT scores. The outcome measures were analyzed using linear mixed‐effects model (LMM).

**Results:**

LMM analysis showed that, compared with conventional intravesical instillation, acupoint application was associated with significant reductions in the total number of voiding episodes within 24 h, daytime voiding frequency, episodes of urinary urgency, and episodes of dysuria following intravesical instillation (all *p* < 0.05). VAS scores and nocturnal voiding frequency also showed decreasing trends, although the differences were not statistically significant (*p* > 0.05). Compared with conventional intravesical instillation, acupoint application resulted in a significant improvement in the physical functioning score of the EORTC QLQ‐C30 (*p* < 0.05). Improvements were also observed in global health status and the role, emotional, cognitive, and social functioning domains; however, these differences did not reach statistical significance (*p* > 0.05). With respect to the symptom domains, scores for fatigue, pain, insomnia, and appetite loss also showed trends toward improvement after acupoint application, but the differences were not statistically significant (*p* > 0.05). No statistically significant differences were observed in any functional domain of the FACT scale between acupoint application and conventional intravesical instillation (*p* > 0.05).

**Conclusion:**

This exploratory study provides preliminary evidence that TCM acupoint application may have the potential to alleviate LUTS in patients after surgery for bladder cancer, while also showing a trend toward improving patients’ quality of life. These findings provide preliminary evidence to support the feasibility of subsequent studies, optimization of study design, and estimation of sample size for future trials.

**Trial Registration:** Chinese Clinical Trial Registry: ChiCTR2300074736

## 1. Introduction

Bladder cancer is one of the most common malignancies of the genitourinary system and represents a significant threat to human health, ranking 10th in global cancer incidence. Approximately 75% of newly diagnosed bladder cancer cases are non–muscle‐invasive bladder cancer (NMIBC), and the proportion of NMIBC is even higher among patients younger than 40 years [1, 2]. Transurethral resection of bladder tumor (TURBT) is the standard first‐line approach for both diagnosis and treatment of NMIBC. Postoperative intravesical instillation therapy, tailored according to tumor pathological characteristics, is an essential adjuvant strategy to prevent tumor recurrence and progression. However, up to 50%–60% of patients experience lower urinary tract symptoms (LUTS), such as urinary urgency, frequency, dysuria, or difficulty in urination, within 48 h after instillation. These symptoms are particularly pronounced during the induction phase and significantly impair patients’ quality of life [3, 4].

Guidelines from the European Association of Urology (EAU) recommend that LUTS associated with intravesical instillation may be managed with oral medications such as antibiotics, tamsulosin, solifenacin, mirabegron, and oxybutynin [5, 6]. In addition, some studies have shown that daily oral administration of traditional Chinese medicine (TCM) formulations that tonify qi and blood can significantly alleviate postinstillation symptoms, including urgency, frequency, and dysuria [7, 8]. However, oral medications require hepatic and renal metabolism, often necessitate prolonged treatment courses, and may cause adverse effects such as dry mouth, constipation, and increased cardiovascular risk. Moreover, the use of antibiotics may interfere with the efficacy of Bacillus Calmette–Guérin (BCG) therapy, thereby limiting their clinical application [9].

Acupoint application, a form of external therapy in TCM, delivers drugs transdermally, thereby avoiding hepatic and biliary metabolism and reducing systemic toxicity. Herbal compounds applied to acupoints can stimulate the skin and meridians, promote local blood circulation, and enhance drug absorption, resulting in significant pharmacological effects [10, 11]. LUTS following intravesical instillation are primarily caused by irritation of the bladder mucosa by instilled agents. Based on the core TCM principle of “preventive treatment of disease,” acupoint application administered prior to instillation may offer a preventive strategy. Previous studies have demonstrated that acupoint application can alleviate urgency and reduce nocturia in patients with overactive bladder (OAB), as well as improve LUTS after transurethral prostatectomy [12, 13]. However, the underlying mechanisms of its therapeutic effects remain unclear.

Building on exploratory studies of electroacupuncture for female stress urinary incontinence conducted by the team of Zhishun Liu, which informed the selection of acupoints and treatment duration, subsequent large‐scale, multicenter randomized controlled trials provided high‐quality evidence supporting the efficacy of electroacupuncture in reducing urinary leakage [14, 15]. Inspired by this approach, we conducted an exploratory, randomized, single‐blind, crossover study to evaluate the efficacy of TCM acupoint application administered prior to intravesical instillation in alleviating postinstillation LUTS. This study aims to provide preliminary evidence for the feasibility of this intervention and to inform the design, risk management, and sample size estimation of future large‐scale, multicenter trials.

## 2. Materials and Methods

### 2.1. Study Design and Randomization

This was a randomized, single‐blind, crossover, exploratory study conducted in patients undergoing intravesical instillation during the induction phase. Random numbers (“1–40”) were generated using Microsoft Excel by a data analyst. Odd numbers were assigned to Group A, and even numbers to Group B. Group allocation labels (“Group A” or “Group B”) were sealed in sequentially numbered, opaque envelopes. After obtaining written informed consent, participants were enrolled sequentially and assigned to groups according to the corresponding numbered envelope.

Patients in Group A received conventional intravesical instillation during the first session and instillation following acupoint application during the second session. Patients in Group B received instillation following acupoint application first, followed by conventional instillation. Based on the herbal ingredients used for the acupoint application, a 1‐week washout period was implemented between the first and second intravesical instillation periods [14, 15]. Because no placebo patch was used and the acupoint application intervention was readily identifiable, blinding of the participants and healthcare professionals administering the intervention was not feasible. Therefore, only the data analyst was blinded to treatment allocation during the statistical analysis. This study was approved by the Institutional Ethics Committee (Approval No. 2023‐R480).

### 2.2. Participants

Patients who underwent TURBT and subsequently received intravesical instillation at the outpatient department of Urology, Second Hospital of Hebei Medical University, between August 2023 and August 2024 were enrolled.

#### 2.2.1. Inclusion Criteria

Inclusion criteria were defined as follows: (1) patients after TURBT; (2) pathological diagnosis of bladder cancer requiring regular intravesical instillation; (3) patients in the induction phase of instillation therapy; (4) age ≥ 18 years; and (5) provision of written informed consent.

#### 2.2.2. Exclusion Criteria

Exclusion criteria were defined as follows: (1) inability to complete instillation therapy due to severe adverse reactions; (2) change of instillation agent during treatment; (3) indwelling urinary catheter; and (4) communication disorders.

### 2.3. Interventions

#### 2.3.1. Conventional Intravesical Instillation

After bladder emptying, a size 10 sterile catheter was inserted under aseptic conditions, and the prepared instillation solution was slowly infused into the bladder. The catheter was then removed, and the solution was retained in the bladder.

#### 2.3.2. Intravesical Instillation Following Acupoint Application

Acupoint application was performed prior to conventional instillation. Herbal patches were applied to Zhongji (RN3), Guanyuan (RN4), Qihai (RN6), Zusanli (ST36), Yinlingquan (SP9), and Sanyinjiao (SP6) (Figure 1).

**FIGURE 1:**
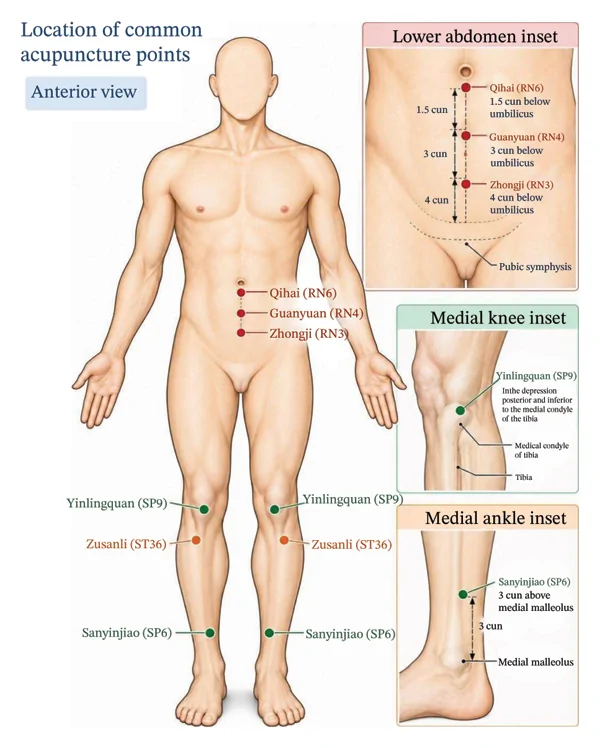
Locations of the acupuncture points used in the study.

Preparation of the herbal paste: equal proportions (1:1) of Lindera aggregata (Wu Yao), Aucklandia lappa (Mu Xiang), Ligusticum chuanxiong (Chuanxiong), Curcuma zedoaria (E Zhu), Liquidambar formosana (Lu Lu Tong), Phellodendron amurense (Huang Bai), Cinnamomum cassia (Rou Gui), and Syzygium aromaticum (Ding Xiang) were ground into powder and sieved. The powder (g) was mixed with white vinegar (mL) at a ratio of 1–1.5:2–3 to form a viscous paste, which was then applied onto patches (6 mm × 6 mm) with an anti‐leakage ring.

All acupoint applications were performed by an experienced TCM physician in the outpatient clinic. Patients were then referred to the instillation room, where the same experienced urologist performed the intravesical instillation. The acupoint patches were retained for 6 h.

### 2.4. Outcome Measures

#### 2.4.1. Primary Outcomes

Primary outcomes included pain severity assessed by the visual analog scale (VAS) and urinary symptoms recorded in a 24‐h voiding diary, including frequency, urgency episodes, and dysuria episodes.

The VAS score ranged from 0 to 10, where 0 indicated no pain and 10 indicated unbearable pain. Participants selected a score according to their perceived pain intensity. VAS was assessed at 6 h after instillation.

The 24‐h voiding diary recorded voiding frequency and associated symptoms, beginning immediately after instillation and completed within 24 h.

#### 2.4.2. Secondary Outcomes

Secondary outcomes included quality of life assessed by the European Organization for Research and Treatment of Cancer Quality of Life Questionnaire‐Core 30 (EORTC QLQ‐C30) and the Functional Assessment of Cancer Therapy–Bladder (FACT‐BL) [16, 17]. In the EORTC QLQ‐C30, higher scores on the functional scales and global health status indicate better functioning or quality of life, whereas higher scores on the symptom scales/items indicate a greater symptom burden. The EORTC QLQ‐C30 was administered 1 week after intravesical instillation, when participants returned to the hospital for their subsequent instillation treatment. FACT‐BL consists of the FACT‐G and the bladder cancer–specific module (BICS), with higher scores indicating better quality of life. FACT‐BL was also collected 1 week after instillation.

### 2.5. Data Collection and Quality Control

Baseline data, including age, sex, surgery date, number of instillations, and instillation agents, were collected upon enrollment. After the first instillation, participants were provided with paper versions of the VAS and voiding diary, along with standardized, non‐leading instructions regarding completion time, procedures, and precautions. At 6 h postinstillation, participants were reminded to remove the acupoint patches, complete the VAS assessment, and record their scores [4, 18]. To ensure data accuracy and reliability, participants were required to submit images of completed VAS forms via WeChat. They were also instructed to complete and submit the 24‐h voiding diary in the same manner. Researchers reviewed submissions for completeness and clarified any inconsistencies with participants to minimize errors. If, during the communication process, a participant confirmed that there were concerns regarding the accuracy or validity of the data but declined further communication for clarification, the data were considered invalid and excluded from the final statistical analysis. Before the second instillation, the previous week’s paper records were collected, and EORTC QLQ‐C30 and FACT‐BL assessments were completed on‐site. Questionnaire data were considered invalid and excluded from the statistical analysis if logical inconsistencies were identified between responses to reverse‐scored items and positively worded items. Data collection during the second session followed the same procedures. Participants were instructed to contact researchers immediately if they experienced skin discomfort (e.g., itching or pain) at the acupoint application site. If skin reactions such as redness or rash occurred, appropriate management was provided by the TCM physician.

### 2.6. Statistical Analysis

Statistical analyses were performed using SPSS Statistics, Version 27.0 (IBM Corp., Armonk, NY, USA). For baseline characteristics, continuous variables with skewed distributions were summarized as medians with interquartile ranges (IQRs), and between‐group comparisons were performed using the Mann–Whitney *U* test. Categorical variables were presented as frequencies or percentages and compared between groups using Fisher’s exact test.

This study adopted a randomized crossover design, in which each participant received both acupoint application combined with intravesical instillation and intravesical instillation alone. Therefore, observations obtained from the two treatment periods within the same participant were correlated. To take full advantage of the within‐subject control inherent in the crossover design and to avoid potential bias associated with conventional independent‐sample analyses, linear mixed‐effects model (LMM) was used to analyze the study outcomes. Treatment, treatment period, and treatment sequence were included in the models as fixed effects. Specifically, the treatment effect was included to evaluate the primary effect of acupoint application compared with conventional intravesical instillation; the period effect was included to assess potential temporal effects between the first and second treatment periods; and the sequence effect was included to determine whether the order of treatment administration (Group A: conventional intravesical instillation ⟶ acupoint application; Group B: acupoint application ⟶ conventional intravesical instillation) influenced the outcomes.

Meanwhile, participant was included in the model as a random effect, with a participant‐specific random intercept specified to account for the within‐participant correlation between repeated measurements obtained across different treatment periods. The specific model was as follows:

(1) Yij=β0+β1Treatmentij+β2Periodij+β3Sequenceij+ui+eij,

where *Y*_ij_ denotes the observed outcome for participant *i* during treatment period *j*; Treatment_ij_ indicates whether the participant received the acupoint application intervention; Period_ij_ represents the first or second treatment period; Sequence_*i*_ represents the treatment sequence to which the participant was assigned; *X*_*i*_ represents the prespecified covariates, including age, sex, intravesical medication, and pathological grade; *b*_*i*_ represents the participant‐specific random effect; *e*_ij_ represents the residual error; and *β* represents the model parameters.

Model parameters were estimated using an appropriate likelihood‐based estimation method. Outcomes were evaluated using estimated marginal means, and the adjusted mean differences between the acupoint application and conventional intravesical instillation conditions, together with their 95% confidence intervals (CIs) and corresponding *p* values, were reported. All primary and secondary outcomes, including pain intensity assessed using the VAS, voiding‐related outcomes (daytime voiding frequency, nocturnal voiding frequency, total voiding frequency, episodes of dysuria, and episodes of urinary urgency), EORTC QLQ‐C30 scores, and FACT scores, were analyzed using LMM. All statistical tests were two‐sided, and a *p* value < 0.05 was considered statistically significant. Given the exploratory nature of this study, no adjustment for multiple comparisons was performed; therefore, the *p* values should be interpreted as exploratory rather than confirmatory.

## 3. Results

### 3.1. Baseline Characteristics

A total of 40 patients who met the inclusion and exclusion criteria were enrolled between August 2023 and August 2024. Patients were randomly assigned to Group A (*n* = 20) or Group B (*n* = 20). In Group A, two patients were lost to follow‐up due to receiving treatment at a nearby hospital, one withdrew due to refractory hematuria, and three withdrew due to refusal to complete questionnaires. In Group B, two patients withdrew due to mild allergic reactions at the acupoint application sites. The overall attrition rate was 20%, and 32 patients ultimately completed the study (participant flow is shown in Figure 2). Baseline characteristics are presented in Table 1. There were no statistically significant differences between Group A and Group B in terms of age, sex, educational level, instillation agent, or pathological grade.

**FIGURE 2:**
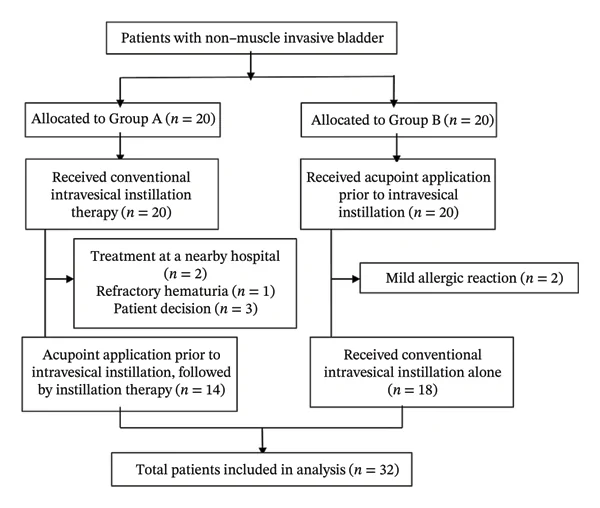
Consort diagram.

**TABLE 1:** Baseline characteristics of the patients.

Clinical characteristics	Total (*n* = 32)	Group A (*n* = 14)	Group B (*n* = 18)	*p* value
Age (years)	66.50 (61.00, 70.75)	67.50 (61.00, 75.50)	65.00 (59.75, 70.25)	0.377
Gender [*n* (%)]				1.00
Male	25 (78.1)	11 (78.6)	14 (77.8)	
Female	7 (21.9)	3 (21.4)	4 (22.2)	
Education level [*n* (%)]				0.700
University or above	5 (15.6)	1 (7.1)	4 (22.2)	
High school/technical school	11 (34.3)	6 (42.9)	5 (27.8)	
Middle school	7 (21.9)	3 (21.4)	4 (22.2)	
Primary school or below	9 (28.1)	4 (28.6)	5 (27.8)	
Medical insurance [*n* (%)]				0.850
Provincial/municipal insurance	20 (62.5)	8 (57.1)	12 (66.7)	
Urban resident insurance	2 (6.3)	1 (7.1)	1 (5.6)	
New rural cooperative medical scheme	10 (31.3)	5 (35.7)	5 (27.8)	
Instillation drug [*n* (%)]				0.425
Epirubicin	7 (21.9)	2 (14.3)	5 (27.8)	
Pirarubicin	15 (46.9)	6 (42.9)	9 (50.0)	
BCG	10 (31.3)	6 (42.9)	4 (22.2)	
Pathological grade [*n* (%)]				0.308
High‐grade	17 (53.1)	9 (64.3)	8 (44.4)	
Low‐grade	15 (46.9)	5 (35.7)	10 (55.6)	

*Note:* *p* values represent comparisons between Group A and Group B. Data are presented as median (interquartile range) for age and as number (percentage) for categorical variables.

### 3.2. Effects of Acupoint Application on LUTS

The treatment effect analysis using the LMM model showed that, compared with conventional intravesical instillation, acupoint application was associated with a trend toward a reduction in pain intensity as assessed by the VAS (mean difference: −0.552, 95% CI: −1.139 to 0.036, *p* = 0.065), although the difference did not reach statistical significance (Figure 3). Compared with conventional intravesical instillation, acupoint application significantly reduced daytime voiding frequency (mean difference: −1.690, *p* = 0.003), total voiding frequency (mean difference: −1.956, *p* = 0.005), episodes of urinary urgency (mean difference: −2.250, *p* = 0.017), and episodes of dysuria (mean difference: −2.286, *p* = 0.014). Nocturnal voiding frequency also showed a decreasing trend, but the difference was not statistically significant.

**FIGURE 3:**
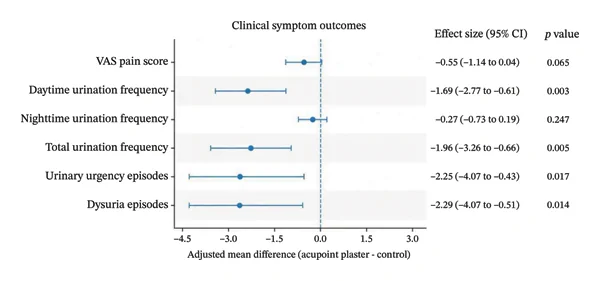
Therapeutic effects of acupoint application on pain and urinary symptoms in patients.

### 3.3. Effects of Acupoint Application on Quality of Life

LMM analysis showed that, compared with conventional intravesical instillation, acupoint application significantly improved the physical functioning score (mean difference: 4.365, 95% CI: 0.423–8.307, *p* = 0.031) (Figure 4). In addition, improvements were observed in global health status and in the role, emotional, cognitive, and social functioning domains, although these differences did not reach statistical significance. With regard to the symptom domains, scores for fatigue, pain, insomnia, and appetite loss also showed trends toward improvement following acupoint application, but none of these differences were statistically significant.

**FIGURE 4:**
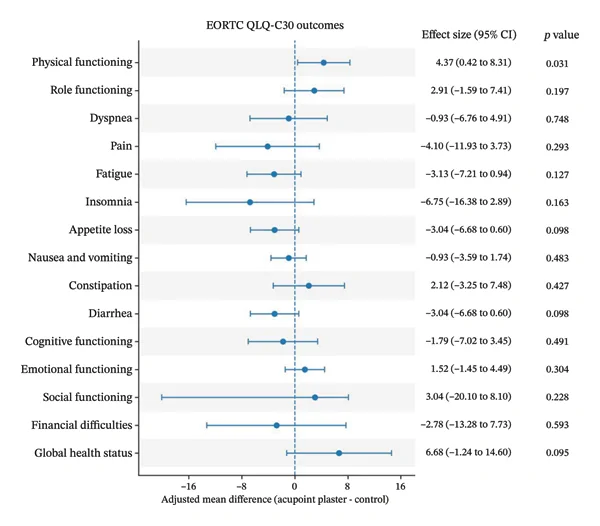
Effects of acupoint application on EORTC QLQ‐C30 scores in patients.

LMM analysis of the FACT scores showed that no statistically significant differences were observed between acupoint application and conventional intravesical instillation across any of the functional domains (Figure 5). Nevertheless, several domains showed trends toward improvement, including social/family well‐being (mean difference: 1.118), emotional well‐being (mean difference: 1.037), and functional well‐being (mean difference: 0.846), all of which demonstrated positive changes.

**FIGURE 5:**
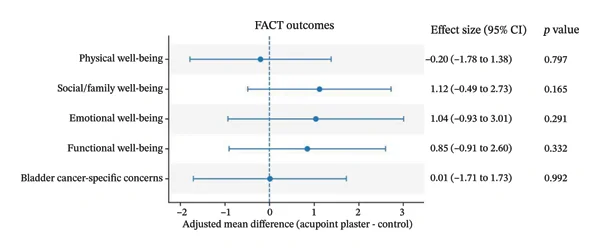
Effects of acupoint application on FACT domain scores.

### 3.4. Effects of Treatment Period and Treatment Sequence on Outcomes in the Crossover Trial

The effects of treatment period and treatment sequence in the crossover trial were further evaluated (Table 2). No significant period or sequence effects were observed for the major clinical outcomes, including VAS score, daytime and nocturnal voiding frequency, total voiding frequency, episodes of urinary urgency, or episodes of dysuria (all *p* > 0.05). However, significant sequence effects were identified for several secondary quality‐of‐life outcomes, including insomnia and appetite loss in the EORTC QLQ‐C30, as well as the physical, social/family, and functional well‐being domains of the FACT questionnaire (*p* < 0.05).

**TABLE 2:** Assessment of treatment period and sequence effects in the crossover study.

Category	Outcome	Period *p* value	Sequence *p* value
Clinical symptoms	VAS pain score	0.25	0.622
Clinical symptoms	Daytime voiding frequency	0.376	0.227
Clinical symptoms	Nocturnal voiding frequency	0.589	0.827
Clinical symptoms	Total voiding frequency	0.354	0.312
Clinical symptoms	Episodes of urinary urgency	0.407	0.613
Clinical symptoms	Episodes of dysuria	0.807	0.41
EORTC QLQ‐C30	Physical functioning	0.967	0.812
EORTC QLQ‐C30	Role functioning	0.442	0.387
EORTC QLQ‐C30	Dyspnea	0.748	0.501
EORTC QLQ‐C30	Pain	0.891	0.978
EORTC QLQ‐C30	Fatigue	0.983	0.428
EORTC QLQ‐C30	Insomnia	0.362	**0.016**
EORTC QLQ‐C30	Appetite loss	0.713	**0.025**
EORTC QLQ‐C30	Nausea and vomiting	0.483	0.867
EORTC QLQ‐C30	Constipation	0.92	0.729
EORTC QLQ‐C30	Diarrhea	0.713	0.969
EORTC QLQ‐C30	Cognitive functioning	0.491	0.937
EORTC QLQ‐C30	Emotional functioning	0.822	0.491
EORTC QLQ‐C30	Social functioning	0.791	0.705
EORTC QLQ‐C30	Financial difficulties	0.593	0.707
EORTC QLQ‐C30	Global health status	0.852	0.797
FACT	Physical well‐being	0.527	**0.001**
FACT	Social/family well‐being	0.188	**0.001**
FACT	Emotional well‐being	0.924	0.249
FACT	Functional well‐being	0.441	**0.001**
FACT	Bladder cancer–specific module	0.43	0.375

*Note:* The *p* values in this table represent the effects of treatment period and treatment sequence only and should not be interpreted as the treatment effect of acupoint application versus conventional infusion. The treatment effects are shown in Figures 3–5 and Table 3. Bold values indicate statistical significance (*p* < 0.05).

### 3.5. Estimated Marginal Means of Study Outcomes Following Conventional Intravesical Instillation and Acupoint Application Combined With Intravesical Instillation

To further evaluate the magnitude of the treatment effect, estimated marginal means for each outcome were compared between the acupoint application and conventional intravesical instillation conditions (Table 3). Overall, the major LUTS outcomes showed a consistent direction of improvement following acupoint application. The VAS score decreased from 2.369 to 1.817, daytime voiding frequency from 9.762 to 8.071 episodes, total voiding frequency from 12.528 to 10.571 episodes, episodes of urinary urgency from 5.171 to 2.921, and episodes of dysuria from 7.353 to 5.067. Although the differences in VAS score and nocturnal voiding frequency did not reach statistical significance, the major urinary irritation symptoms generally showed consistent numerical reductions, in line with the direction of the estimated treatment effects.

**TABLE 3:** Estimated marginal means of outcome measures and *p* values for treatment effects between conventional infusion and postacupoint application infusion.

Category	Outcome	Adjusted mean: Conventional intravesical instillation	Adjusted mean: Acupoint application	Treatment effect *p* value
Clinical symptoms	VAS pain score	2.369	1.817	0.065
Clinical symptoms	Daytime voiding frequency	9.762	8.071	0.003
Clinical symptoms	Nocturnal voiding frequency	2.766	2.5	0.247
Clinical symptoms	Total voiding frequency	12.528	10.571	0.005
Clinical symptoms	Episodes of urinary urgency	5.171	2.921	0.017
Clinical symptoms	Episodes of dysuria	7.353	5.067	0.014
EORTC QLQ‐C30	Physical functioning	87.566	91.931	0.031
EORTC QLQ‐C30	Role functioning	91.468	94.378	0.197
EORTC QLQ‐C30	Dyspnea	10.582	9.656	0.748
EORTC QLQ‐C30	Pain	18.188	14.087	0.293
EORTC QLQ‐C30	Fatigue	21.252	18.122	0.127
EORTC QLQ‐C30	Insomnia	27.513	20.767	0.163
EORTC QLQ‐C30	Appetite loss	7.672	4.63	0.098
EORTC QLQ‐C30	Nausea and vomiting	3.042	2.116	0.483
EORTC QLQ‐C30	Constipation	8.201	10.317	0.427
EORTC QLQ‐C30	Diarrhea	5.159	2.116	0.098
EORTC QLQ‐C30	Cognitive functioning	88.624	86.939	0.491
EORTC QLQ‐C30	Emotional functioning	86.938	88.459	0.304
EORTC QLQ‐C30	Social functioning	85.582	88.624	0.228
EORTC QLQ‐C30	Financial difficulties	24.603	21.825	0.593
EORTC QLQ‐C30	Global health status	68.386	75.066	0.095
FACT	Physical well‐being	12.518	12.316	0.797
FACT	Social/family well‐being	11.545	12.663	0.165
FACT	Emotional well‐being	18.243	19.28	0.291
FACT	Functional well‐being	11.204	12.051	0.332
FACT	Bladder cancer–specific module	29.569	29.578	0.992

Changes in quality‐of‐life outcomes were comparatively modest. For the EORTC QLQ‐C30, the physical functioning score increased from 87.566 to 91.931 following acupoint application and reached statistical significance. The global health status score also increased from 68.386 to 75.066; however, this finding should be interpreted as a potential trend toward improvement rather than confirmatory evidence. Symptom‐domain scores, including pain, fatigue, insomnia, and appetite loss, generally decreased after acupoint application, although the corresponding effect estimates were associated with substantial uncertainty.

In contrast, estimated marginal means across the FACT domains differed only slightly between the two treatment conditions. These findings suggest that the short‐term benefits of acupoint application may be primarily reflected in the alleviation of urinary irritation symptoms associated with intravesical instillation, whereas the intervention may not be sufficient to produce broad and consistent improvements in overall quality of life over the short term.

## 4. Discussion

Intravesical chemotherapy and immunotherapy have been proven to effectively reduce recurrence and progression of NMIBC after TURBT, and are an essential component of standard treatment [19]. The major advantage of intravesical instillation lies in its local delivery, which minimizes systemic adverse effects. However, direct exposure of the bladder mucosa to chemotherapeutic and immunotherapeutic agents can lead to significant irritation and LUTS [9, 19]. These adverse effects may be associated with multiple mechanisms, including sensitization of bladder afferent nerves induced by instilled agents, inflammation‐triggered hypersensitivity, and disruption of the urothelial barrier permeability [3, 20].

Acupoint application therapy is a form of external treatment in TCM, based on TCM theory and transdermal drug delivery. Acupoints are characterized by a thinner epidermis, dense sensory receptors, and abundant capillaries and nerve endings, which facilitate drug absorption and transmission. Combined with the pharmacological effects of herbal medicine, acupoint stimulation exerts a dual therapeutic effect [10, 11]. The bladder is primarily innervated by the thoracolumbar (T10–L2) and sacral (S2–S4) segments. Previous studies have shown that stimulation of body surface acupoints can exert segmental and systemic regulatory effects on the sympathetic nervous system [21, 22]. By selecting acupoints within these innervated regions, afferent neural signals transmitted to the spinal cord may be modulated through central integration and interaction within these segments. From a neurobiological perspective, stimulation of these acupoints may therefore reduce LUTS following intravesical instillation [23, 24].

The results of this study showed that, compared with conventional intravesical instillation, acupoint application significantly reduced daytime voiding frequency, total voiding frequency, episodes of urinary urgency, and episodes of dysuria within 24 h after intravesical instillation. The selected acupoints in this study—Zhongji (RN3), Guanyuan (RN4), and Qihai (RN6)—are located in the lower abdomen, corresponding anatomically to the bladder region and within the T10–L2 innervation segment [25, 26]. Stimulation of Zhongji has been reported to activate parasympathetic pathways and regulate coordination between the detrusor muscle and urethral sphincter [25, 27]. Zusanli (ST36), Yinlingquan (SP9), and Sanyinjiao (SP6) are located within the L2–S3 segments; stimulation of Sanyinjiao has been shown to inhibit neural impulses from the micturition center and increase urethral resistance [18, 28]. Emerging evidence suggests that targeting ion channels, including transient receptor potential (TRP) channels highly expressed in bladder sensory nerves, can suppress bladder afferent signaling and reduce LUTS [29, 30]. The volatile oil extracted from Lindera aggregata has been shown to downregulate the mechanosensitive ion channel Piezo1, thereby reducing bladder sensitivity and improving LUTS [31]. Extracts from Phellodendron amurense, when dissolved in vinegar, can inhibit smooth muscle contraction induced by neural stimulation, norepinephrine, acetylcholine, and ATP, acting on nonspecific postganglionic sites to relieve urinary symptoms [32, 33].

The herbal formulation used in this study included Curcuma zedoaria, Syzygium aromaticum, Lindera aggregata, Phellodendron amurense, Aucklandia lappa, Ligusticum chuanxiong, Liquidambar formosana, and Cinnamomum cassia. Aucklandia lappa promotes qi circulation and relieves pain, while Ligusticum chuanxiong, combined with Curcuma zedoaria, promotes blood circulation, removes stasis, and alleviates pain. Liquidambar formosana facilitates diuresis and relieves stranguria. Based on the TCM principle of “preventive treatment,” acupoint application prior to intravesical instillation may exert synergistic effects through both herbal permeation and acupoint stimulation, thereby alleviating postinstillation LUTS. The potential of TCM acupoint application to alleviate LUTS following intravesical instillation may be partly attributable to these mechanisms. Curcumin, an active component of *Curcuma zedoaria*, and eugenol, the major pharmacologically active constituent of clove, may exert analgesic effects through modulation of TRP channels [34–36]. Accordingly, the potential improvement in postinstillation dysuria associated with TCM acupoint application may result from the combined effects of acupoint stimulation and transdermal absorption of the herbal constituents. Previous studies have reported that acupoint application with Cinnamomum cassia significantly alleviates urgency and frequency in patients with OAB [12]. A randomized controlled trial conducted in the United States demonstrated that 30 min of acupuncture stimulation prior to intravesical BCG instillation could reduce LUTS [37]. These findings are consistent with the trends observed in the present study.

Considering that the subjects received different intravesical instillation agents, we further incorporated the type of instillation agent and the interaction term between treatment modality and instillation agent into the LMM for exploratory analysis. The model was constructed as follows: $Y_{\text{ij}}=\beta_{0}+\beta_{1}{\text{Treatment}}_{\text{ij}}+{\beta_{2}\text{Period}}_{\text{ij}}+{\beta_{3}\text{Sequence}}_{\text{ij}}+{\beta_{4}\text{Agent}}_{\text{ij}}+\beta_{5}{\text{Treatment}}_{\text{ij}}\times {\text{Agent}}_{\text{ij}}+u_{i}+e_{\text{ij}}$. Herein, agent represented epirubicin, pirarubicin, or BCG. Treatment × agent was used to test whether the therapeutic effect of acupoint application varied across different instillation agents.

The results showed that no significant main effect of instillation agent was observed for nocturnal urinary frequency, daytime urinary frequency, total urinary frequency, urgency episodes, and dysuria episodes (all *p* > 0.05). Meanwhile, the interaction between treatment modality and instillation agent did not reach statistical significance (*p* = 0.123, 0.517, 0.274, 0.119, and 0.263, respectively). These findings indicate that within the sample of this study, there is no statistical evidence that the therapeutic effect of acupoint application differs markedly by the type of intravesical instillation agent. Given the small sample size in each agent subgroup, this result should be interpreted only as an exploratory analysis; see Table 4 for details.

**TABLE 4:** Estimated marginal means of each outcome indicator in patients receiving conventional intravesical instillation versus intravesical instillation following acupoint application.

Category	Outcome indicator	Epirubicin (*n* = 7), adjusted mean difference (95% CI), *p* value	Pirarubicin (*n* = 15), adjusted mean difference (95% CI), *p* value	BCG (*n* = 10), adjusted mean difference (95% CI), *p* value	*p* for main effect of instillation agent	*p* for treatment × instillation agent interaction
Clinical symptoms	Nocturnal urinary frequency	−0.800 (−1.587 to −0.013), *p* = 0.047	+0.208 (−0.436 to 0.851), *p* = 0.514	−0.523 (−1.483 to 0.437), *p* = 0.274	0.797	0.123
Clinical symptoms	Daytime urinary frequency	−2.600 (−4.546 to −0.654), *p* = 0.011	−1.232 (−2.822 to 0.359), *p* = 0.124	−1.347 (−3.721 to 1.027), *p* = 0.255	0.676	0.517
Clinical symptoms	Total urinary frequency	−3.400 (−5.689 to −1.111), *p* = 0.005	−1.024 (−2.896 to 0.847), *p* = 0.272	−1.870 (−4.663 to 0.923), *p* = 0.181	0.656	0.274
Clinical symptoms	Urgency episodes	−4.400 (−7.510 to −1.290), *p* = 0.007	−2.131 (−4.673 to 0.411), *p* = 0.097	+0.728 (−3.066 to 4.523), *p* = 0.697	0.134	0.119
Clinical symptoms	Dysuria episodes	−3.100 (−6.226 to 0.026), *p* = 0.052	−2.970 (−5.525 to −0.415), *p* = 0.024	+0.479 (−3.334 to 4.293), *p* = 0.799	0.080	0.263

*Note:* Data are presented as adjusted mean differences and 95% confidence intervals derived from the linear mixed‐effects model (LMM). The adjusted mean difference was defined as “intravesical instillation following acupoint application minus conventional intravesical instillation.” Therefore, a negative value indicates a reduction in the corresponding symptom or urinary frequency after acupoint application intervention. The *p* value for the main effect of instillation agent reflects the overall difference across different intravesical instillation agents. The *p* value for the treatment × instillation agent interaction was used to examine whether the therapeutic effect of acupoint application differed by instillation agent. Analyses of each drug subgroup were exploratory, and *p* values were not adjusted for multiple comparisons.

Analysis of the period and sequence effects in this crossover trial showed that no significant period effects were observed for any of the study outcomes, indicating that the treatment period itself did not materially influence the outcome assessments. This finding also provides some support for the appropriateness of the crossover design, in which each participant served as their own control. However, significant sequence effects were identified for several secondary quality‐of‐life outcomes in the EORTC QLQ‐C30 and FACT questionnaires. These findings suggest that certain subjective quality‐of‐life measures may be more susceptible to the order of treatment administration, participants’ experiences during the preceding treatment period, temporal changes in disease status, or potential residual effects. Therefore, the numerical changes observed in these outcomes should be interpreted cautiously as exploratory findings and should not, in isolation, be considered confirmatory evidence that acupoint application produces a definitive improvement in quality of life.

### 4.1. Strengths and Limitations

The main strength of this study lies in being the first to apply a noninvasive, external TCM acupoint application as an intervention for patients undergoing intravesical instillation after bladder cancer surgery. The intervention was conveniently administered during routine outpatient visits for instillation, ensuring good feasibility and adherence to the study protocol. A total of four adverse events occurred during the study period, as detailed in Table 5.

**TABLE 5:** Adverse events.

Participant no.	Sequence	Adverse event	Grade	Causal relationship with acupoint application	Led to withdrawal	Outcome
3	B	Mild allergic reaction	Grade 1	Definitely related	YES	Resolved
13	B	Mild allergic reaction	Grade 1	Definitely related	YES	Resolved
14	A	Refractory hematuria	Grade 3	Possibly unrelated	YES	Resolved
39	A	Mild allergic reaction	Grade 1	Definitely related	NO	Resolved

*Note:* The severity of adverse events was graded according to the Common Terminology Criteria for Adverse Events (CTCAE), Version 5.0.

This study has several limitations. First, as an exploratory study conducted at a single center, it was limited by a relatively small sample size. Second, stratification according to intravesical medication, pathological grade, sex, and other potentially relevant factors was not performed. Third, no placebo patch was included in the study design, and the outcomes were primarily patient‐reported; therefore, the potential influences of placebo effects and expectation effects could not be adequately distinguished. Future studies should incorporate the methodological experience gained from the present study to further optimize the study design and provide a foundation for subsequent multicenter studies with larger sample sizes.

## 5. Conclusion

In this exploratory crossover trial, TCM acupoint application showed preliminary potential to improve LUTS in patients undergoing intravesical instillation after bladder cancer surgery, particularly urinary urgency and frequency. A trend toward improvement in quality of life was also observed. This study provides important preliminary evidence supporting the feasibility of this intervention and offers a basis for sample size estimation and methodological refinement in future randomized controlled trials with larger sample sizes and more rigorous designs (e.g., randomized, double‐blind studies). As TCM therapies gain increasing attention as adjunctive treatments for cancer patients, this study offers early insights into the potential role of acupoint application in alleviating postinstillation LUTS in patients with bladder cancer.

## Author Contributions

Lei Shi: conceptualization, methodology, supervision, quality control, data curation, writing–original draft. Cong Chen: data collection, data curation, project administration, formal analysis, writing–original draft. Lian Li: conceptualization, investigation, project administration. Junxiao Chen: project administration, data collection, investigation. Yongtai Zhang: investigation, project administration. Jianhong Li: project administration, data collection, investigation. Zhijuan Xu: conceptualization, data curation. Di Lu: data collection, investigation, data curation, formal analysis. Yingxue Ma: investigation, data collection, project administration. Yuechen Feng: data collection, investigation, data curation. Chenggen Jin: conceptualization, methodology, formal analysis, writing–review and editing. Yaxuan Wang: conceptualization, methodology, writing–review and editing.

## Funding

This study was supported by the Scientific Research Project of the Hebei Provincial Administration of Traditional Chinese Medicine (Grant No. 2025039).

## Ethics Statement

This study was conducted at the Second Hospital of Hebei Medical University in China and was approved by the Institutional Ethics Committee (approval No. 2023‐R480). Written informed consent was obtained from all participants prior to enrollment.

## Conflicts of Interest

The authors declare no conflicts of interest.

## Data Availability

The raw data supporting the findings of this study are available from the corresponding author upon reasonable request.
